# Highly efficient synchronization of sheep skin fibroblasts at G2/M phase and isolation of sheep Y chromosomes by flow cytometric sorting

**DOI:** 10.1038/s41598-020-66905-x

**Published:** 2020-06-18

**Authors:** Yanzhu Yao, Yuanyuan Zhang, Wansheng Liu, Xuemei Deng

**Affiliations:** 1 Key Laboratory of Animal Genetics, Breeding and Reproduction of the Ministry of Agriculture & Beijing Key Laboratory of Animal Genetic Improvement, China Agricultural University, Beijing, 100193 China; 2 Department of Animal Science, Center for Reproductive Biology and Health, College of Agricultural Sciences, Pennsylvania State University, University Park, Pennsylvania 16802 USA

**Keywords:** Chromosome segregation, Kinetochores, Cell culture, Flow cytometry, High-throughput screening

## Abstract

At present, based on whole genome sequencing, sequences and genes annotation of the sheep (*Ovis aries*) Y chromosome are still absent. The isolation of Y chromosomes followed by sequencing has been approved as an effective approach to analyze this complex chromosome in other species. In this study, we established a highly efficient synchronization method for G2/M phase of sheep fibroblasts, which was successfully applied to flow-sorting chromosomes of sheep, with a focus on isolation and sequencing of the ovine Y chromosome. The isolated (~80,000) Y chromosomes were verified by fluorescence quantitative real-time polymerase chain reaction, further confirmed by fluorescence *in situ* hybridization, and amplified by the MALBAC method before next-generation sequencing. The sequence results indicated that 68.90% of reads were Y chromosome-related sequences as they are homologous to the bovine Y chromosome. The remaining 31.1% of reads were aligned to the sheep reference genome, including 13.57% reads to chromosome X and 6.68% to chromosome 17. Importantly, the paired-end reads that are properly aligned to the bovine Y sequence assembly accounted for 46.49%, indicating the success in the ovine Y chromosome isolation and the high quality of the Y chromosome sequences. This study not only set up a foundation for future sequencing, assembly and annotation of the ovine Y chromosome, but also provide a validated approach to overcoming difficulties in sequencing Y chromosome in other mammalian species.

## Introduction

Flow cytometric sorting of chromosomes is considered a powerful tool for chromosome research due to its ability to isolate particular chromosomes with good purity and quantities. The advantage of particular chromosome sequencing is that it can greatly simplify data analysis and reduce interference, as compared to complex genome sequencing. Although genomic sequences of hundreds of species have been published in recent years with the development of next- and third-generation sequencing, the genome sequencing quality for some species still needs to be improved, especially with respect to sex chromosome sequences. However, based on whole genome sequencing, only a few Y chromosomes of all completed genome sequences have been fully sequenced and annotated due to challenges associated with the sequencing and assembly of highly repetitive sequences and nearly identical palindromic regions^1^. For instance, only 18 species completed their Y chromosome assemblies among 98 mammalian species sequenced^2^. The Y chromosome presenting only in males and passaged uniquely along the paternal lineage has many important functions that have not been studied outside of model organisms^2^. Obtaining Y chromosome sequences is important to understand the intricacies of vertebrate genome function and evolution. The traditional method for obtaining Y chromosome sequences was based on bacterial artificial chromosomes and sequencing^2–4^. However, this approach has some disadvantages such as being time-consuming, laborious, and expensive. Although next-generation sequencing (NGS) of the whole genome is faster and cheaper due to technological advances, identifying contigs from the Y chromosome can be challenging. Compared to that with whole genome sequencing, enrichment of Y chromosomes before sequencing might simplify data analysis and reduce sequencing costs. Flow cytometric sorting is attractive because of its capacity to purify single chromosomes in large numbers. Moreover, in recent years, sequencing technology has advanced to permit the sequencing of single chromosomes of interest isolated by flow cytometric sorting. This method has been successfully applied in humans^5,6^, gorillas^1^, Chinese hamsters^7^, and some plant species^8–11^. To date, this application has not been reported with domestic animals. The Y chromosomes of many domestic animals, including the sheep Y chromosome, have not been fully assembled and characterized. In this study, we isolated and enriched sheep Y chromosomes by flow cytometric sorting and performed NGS of sorted Y chromosomes, which laid a foundation for the sequencing, assembly, and analysis of the sheep Y chromosome.

Chromosomes with different sizes and AT/GC contents can be separated by flow cytometric sorting after staining with two specific fluorescent dyes (Hoechst 33258-AT specific binding and chromomycin A3-GC specific binding)^12,13^. However, chromosomes with small differences in size and GC content are not easily segregated. The so-called bivariate flow karyotype is generated based on the fluorescence intensity of the two dyes, and the purity of flow-sorted chromosomes can be greater than 95% with high-quality samples and favorable conditions^12,14^. Further, the flow cytometric sorting of chromosomes has been applied to DNA hybridization, DNA libraries, physical mapping, and chromosome sequencing^12^.

The quality of chromosome samples is critical in chromosome flow cytometric sorting^15,16^. A high-quality chromosome sample contains a large number of single chromosomes in a suitable chromosome isolation buffer. For mammals, chromosome samples are usually prepared from metaphase cells. To obtain a large number of metaphase cells in a short time, cells need to be synchronized in the G2/M phase of the cell cycle. Although the preparation of chromosome suspensions from mammalian cell lines^17,18^, primary cells^19,20^, and peripheral blood lymphocytes^21,22^ has been reported, conditions for cell-cycle synchronization differ among species, and this is especially the case when primary cells differ.

Just like most of mammalian species, the sequences of the ovine-Y chromosome are not available. In this study, we established a highly efficient synchronization method for G2/M phase of sheep fibroblasts suitable for sheep chromosomes flow sorting. We successfully isolated and enriched ovine Y chromosomes by flow cytometric sorting and performed NGS. The purity of sorted sheep Y chromosome was analyzed using NGS data for the first time. This study demonstrates the potential application of flow cytometry for genome and chromosome research and lays a foundation for future sequencing and annotation of the ovine Y chromosome.

## Results

### Establishment, purification, and culture of sheep primary skin fibroblasts

**Figure 1:**
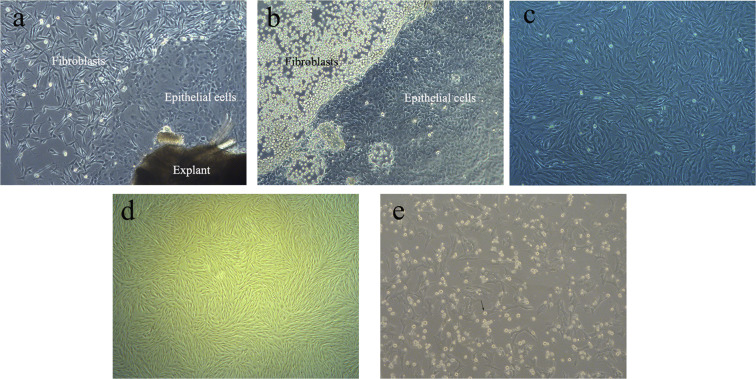
Culture of sheep fibroblasts (100×). (**a**) Primary cells from an explant of ram ear. (**b**) After treating with 0.1% trypsin for 2 min, fibroblasts became round before epithelial cells. (**c**) Fibroblasts at 70–80% confluency. (**d**) Fibroblasts at 90–100% confluency. (**e**) Fibroblasts with a rounded shape were mitotic (black arrows) after treatment with nocodazole.

Epithelial cells around the explants were first seen at 48 h in the primary skin tissue culture, and primary skin fibroblasts were observed on the outer layer of epithelial cells on day 5 (Fig. 1a). Two minutes after short trypsinization with 0.1% trypsin application, fibroblasts became round, before epithelial cells, indicating that fibroblasts were more easily digested than epithelial cells (Fig. 1b). No epithelial cell colonies were observed after three passages, and fibroblasts were the major cells observed under a microscope (Fig. 1c,d).

### Cell-cycle synchronization

To obtain a large number of metaphase cells for the isolation of chromosomes, the synchronization of sheep skin fibroblasts at G2/M phase was performed.

### Observation of mitotic activity in sheep fibroblasts during recovery from double thymidine block treatment

**Figure 2:**
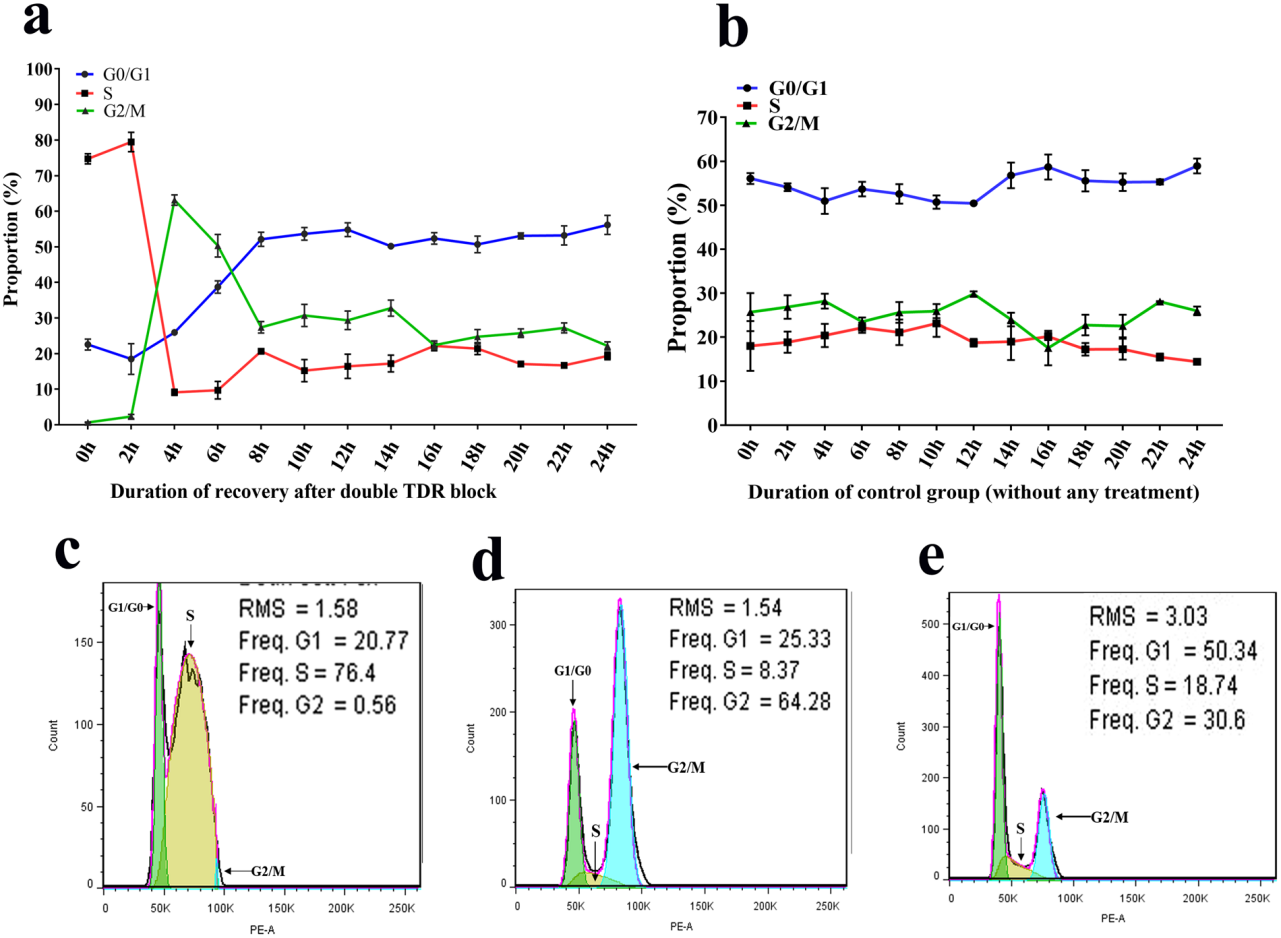
Observation of mitotic activity in sheep fibroblasts. (**a**) Observation of mitotic activity in sheep fibroblasts during recovery after double thymidine (TDR) block treatment (16–18 h TDR treatment followed by 8–10 h recovery and 16–18 h TDR treatment). (**b**) Observation of mitotic activity in sheep fibroblasts without any treatment. (**c**) Flow karyotype histograms of sheep fibroblasts with the smallest proportion of G2/M cells at 0 h of recovery after double thymidine (TDR) block treatment. (**d**) Flow karyotype histograms of sheep fibroblasts with the largest proportion of G2/M cells at 4 h of recovery after double thymidine (TDR) block treatment. (**e**) Flow karyotype histograms of sheep fibroblasts with the largest proportion of G2/M cells at 12 h of recovery in the control group.

After double thymidine (2 mM) block, samples were taken regularly at 2-h intervals for up to 24 h during the recovery. At 0 h of recovery, 74.73 ± 1.18% of the cells were in S phase, while only 0.66 ± 0.07% of cells were in G2/M phases (Fig. 2a,c). Mitotic activity of the fibroblasts occurred rapidly within the next 2–4 h (Fig. 2a). The largest proportion of G2/M cells was 63.14 ± 1.18% obtained at 4 h (Fig. 2a,d), which was significantly higher (*P* < 0.05) than the proportion seen at 0 h and 2 h. During recovery after double TDR block, the increased proportion of cells in G2/M phase was up to 62.47% (Fig. 2a). Meanwhile, the largest proportion of G2/M cells in the control group (fibroblasts without any treatment) was 29.99 ± 0.49% at 12 h (Fig. 2b,e). Thus, we clearly observed the mitotic activity of sheep fibroblasts during recovery from double thymidine block treatment, wherein the proportion of cells in each phase had changed greatly (Fig. 2a). However, in the group of cells that did not receive treatment, the proportion of cells in each phase was only marginally changed (Fig. 2b).

### Effect of nocodazole on sheep fibroblasts

**Figure 3:**
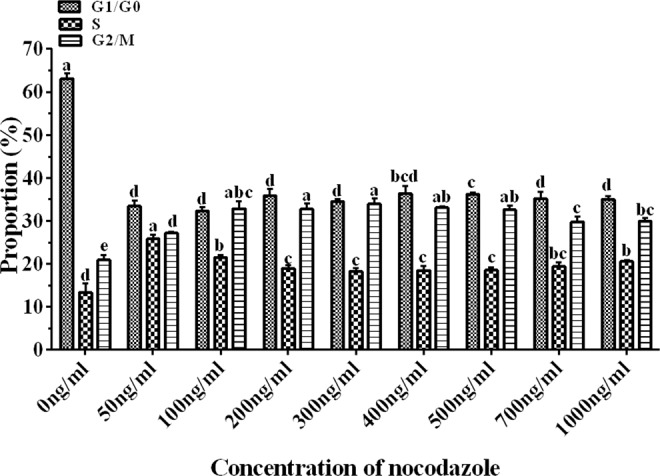
Effect of nocodazole on sheep fibroblasts. The effect of different concentrations of nocodazole on the proportion of cells arrested at different phases (G0/G1, S, and G2/M) of the cell cycle. Bars in the same color with different letters differed significantly (*P* < 0.05).

After treatment with nocodazole, a proportion of cells was observed with a rounded shape, and these were mitotic cells (Fig. 1e). The effect of varying concentrations of nocodazole on the proportion of cells arrested at the G2/M stage is shown in Fig. 3. The percentages of G2/M phase cells in all the groups treated with nocodazole were significantly higher than that of control group (0 ng/ml). And the percentage of G2/M phase cells in the groups treated with nocodazole at concentrations of 100, 200, 300, and 500 ng/mL was not significantly different, but the percentages of G2/M phase cells in all of these groups were significantly higher than those in cells treated with 50 and 700 ng/mL of nocodazole (*P* < 0.05, Fig. 3). The percentage of G2/M phase cells in the group treated with nocodazole at a concentration of 1000 ng/mL was significantly higher than that in cells administered 50 ng/mL of treatment but was significantly lower than that with the other concentrations of nocodazole, except for 700 ng/mL (*P* < 0.05). Thus, 100 ng/mL nocodazole was used for further experiments and for the isolation of metaphase chromosomes in following work (Fig. 3).

### Double thymidine block and colchicine/nocodazole treatment-based synchronization

**Figure 4:**
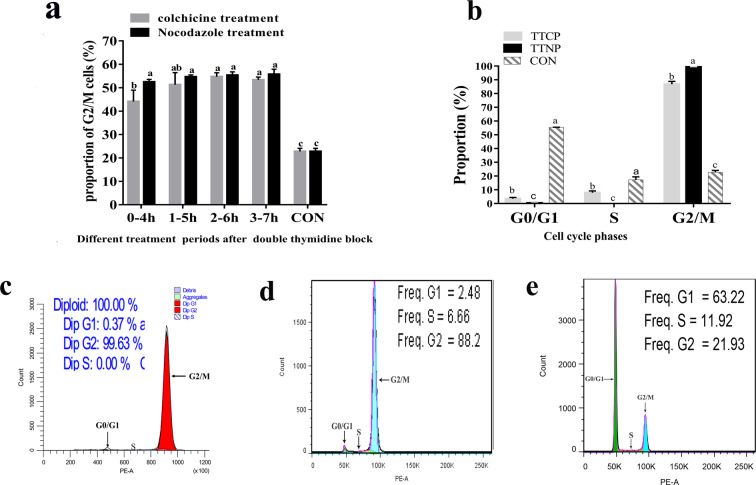
Use of a double thymidine block and colchicine/nocodazole treatment as a cell synchronization method. (**a**) Percentages (mean ± SEM) of cells arrested at the G2⁄M phase in different arresting periods, after treatment with a double thymidine (TDR) block and colchicine/nocodazole. CON: control group, which includes cells without any treatment. (**b**) Percentages (mean ± SEM) of cells arrested at different phases (G0/G1, S, and G2/M) of the cell cycle in different synchronization groups. TTCP: cells collected after patting dishes for double thymidine block and colchicine treatment. TTNP: cells collected after patting dishes for double thymidine block and nocodazole treatment method. CON: cells without any treatment. (**c**) Flow karyotype histograms of cells collected after double thymidine block and nocodazole treatment. (**d**) Flow karyotype histograms of cells collected after double thymidine block and colchicine treatment. (**e**) Flow karyotype histograms of cells without any treatment. Bars with different letters differed significantly (P < 0.05).

Cells were treated with colchicine/nocodazole at varying time intervals after the double thymidine block. There was no significant difference in proportion of cells in the G2/M phase among treatment periods, except for in the colchicine treatment group at 0–4 h (*P* > 0.05, Fig. 4a). However, the proportion of G2/M phase cells was significantly higher in all colchicine/nocodazole treatment groups compared to that of the control group (cells without any treatment; *P* < 0.05, Fig. 4a). For cells treated with the double thymidine block and colchicine/nocodazole treatment, more than 50% were arrested in the G2/M phase. The proportion of G2/M phase cells (designated TTNP) collected after patting dishes for the double thymidine block and nocodazole treatment method was significantly higher than that (designated TTCP) collected after patting dishes based on the colchicine treatment method (*P* < 0.05, Fig. 4b). Further, 99.63% of cells collected after double thymidine block and nocodazole treatment were at the G2/M phase of the cell cycle (Fig. 4c), whereas 86.81 ± 1.70% of the cells collected after double thymidine block and colchicine treatment were at the G2/M phase (Fig. 4d). The proportion of G2/M phase cells in the control group (cells without any treatment) was 22.76 ± 1.14% (Fig. 4a,e), which was significantly lower than that of cells treated with double thymidine block and nocodazole/colchicine (*P* < 0.05, Fig. 4a). Thus, double thymidine block and nocodazole treatment-based synchronization was selected for the accumulation of metaphase cells. In addition, this procedure resulted in a metaphase frequency of 99.63% (Fig. 4c).

### Isolation of metaphase chromosomes

**Figure 5:**
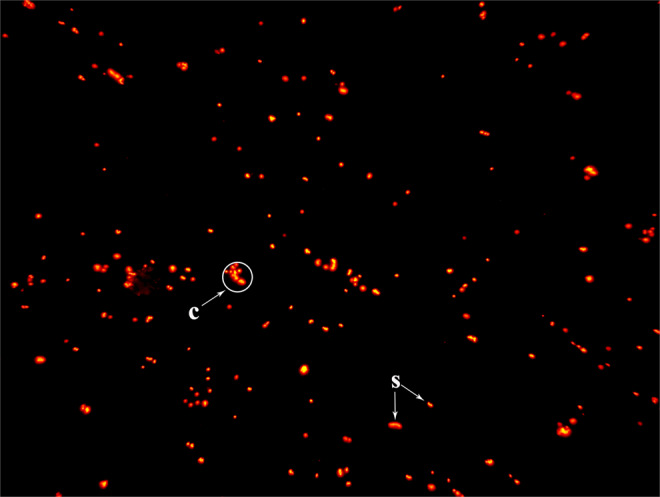
Chromosome suspension stained with PI (propidium iodide) (400×). Large numbers of “single chromosomes” (s) and a few non-dispersed clusters of chromosomes (c) were observed in the chromosome suspension under a microscope. Not all single fluorescent dots were “single floating chromosomes” and there were a few non-dispersed clusters of chromosomes in the chromosome suspension.

Single floating chromosomes were checked under a fluorescence microscope, and the images of “single floating chromosomes” are shown in Fig. 5. There were a large number of “single floating chromosomes” and a few non-dispersed clusters of chromosomes (Fig. 5) in the suspension after treating the swollen cells with polyamine isolation buffer.

### Flow karyotype analysis of ovine chromosomes and isolation of Y chromosomes

**Figure 6:**
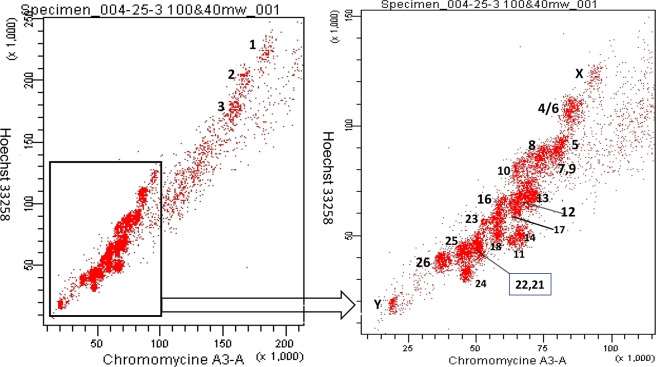
Bivariate flow karyotype of sheep fibroblasts from a ram. (**a**) Flow karyotype including the large metacentric sheep chromosomes. (**b**) Amplification of the lower region of image (**a**).

The bivariate flow karyotypes of sheep, based on the relative Hoechst 33258 and Chromomycin A3 fluorescent intensity, are shown in Fig. 6. Approximately 80,000 Y chromosomes were collected.

### Verification of the sorted ovine Y chromosomes

#### WGA of the sorted ovine Y chromosomes

To obtain a sufficient amount of Y DNA, the MALBAC method was applied for the WGA of flow-sorted ovine Y chromosomes. The fragment lengths of the WGA products ranged from 100 bp to> 2000 bp (Supplementary Fig. S1 and Supplementary Table S1). The amplified Y DNA was used for further FqRT-PCR, FISH, and NGS.

#### FqRT-PCR verification of the sorted ovine Y chromosomes

FqRT-PCR with the Y chromosome-specific gene primers was performed using amplified Y DNA as a template. The Ct value of each pair of Y-specific primers using amplified Y chromosomal DNA as the template was significantly lower than that with the corresponding pair of primers using ovine genomic DNA as a template (*P* < 0.05, Supplementary Table S2 and Supplementary Figure S2). This result indicated that the ovine Y chromosome was indeed enriched by our flow cytometric sorting procedure and that the FqRT-PCR amplification of sorted Y DNA was successful.

#### FISH verification of the sorted ovine Y chromosomes

**Figure 7:**
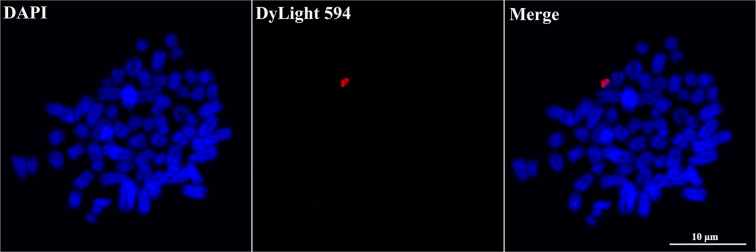
Verification of flow-sorted Y chromosomes by fluorescence *in situ* hybridization (FISH). Positive signals were present on Y chromosome. Scale bar represents 10 μm.

To further confirm the identity and coverage of the Y chromosomes, FISH was performed on metaphase spreads of sheep primary skin fibroblasts, using paint probes prepared with amplified Y chromosomal DNA. Positive signals were present on Y chromosome (Fig. 7).

#### NGS verification of the sorted ovine Y chromosomes

**Figure 8:**
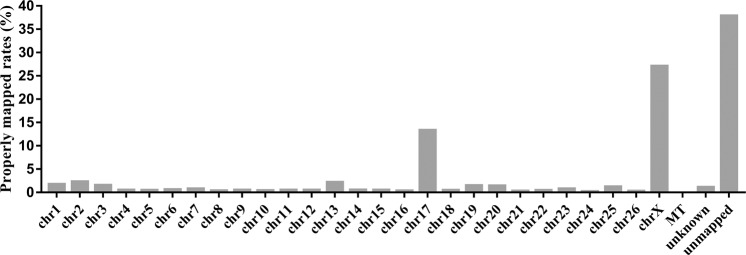
NGS results of flow sorted sheep Y chromosome. The X-axis shows each analyzed chromosome and the Y-axis shows the proportion of NGS reads of flow sorted sheep Y chromosome properly paired to chromosomes.

NGS of WGA DNA produced 33,015,480 reads and 9,893,027,006 bases (∼9.80 Gb). Further, 31.10% of the reads were properly aligned to the sheep reference genome (OAR v4.0). NGS and sequence analysis indicated that 68.90% of reads were Y chromosome-related sequences as they had homologous sequences in the ovine Y chromosome. The remaining 31.1% of reads were aligned to the ovine reference genome, including 13.57% of reads aligned to the X chromosome and 6.68% aligned to chromosome 17. The rate of properly paired reads aligned to each chromosome is shown in Fig. 8. In addition, only a very small number of reads (<1.20%) mapped to each remaining chromosome (chromosome 1–16, chromosome 18–26) and unknown reference sequences of the ovine genome (Fig. 8 and Supplementary Table S3). Additionally, 63.28% of reads mapped to the Y chromosome sequence of cattle and 46.49% were correctly aligned. The fact that 63.28% of reads mapped to the cattle Y chromosome sequence and that 68.90% of the NGS reads were Y chromosome-related sequences indicated that the flow-sorted chromosome fragments mainly originated from the ovine Y chromosome.

## Discussion

Flow cytometric sorting has become an attractive and powerful tool in chromosomes genomics due to its ability to isolate individual chromosomes in large quantities with a high degree of purity. Compared to microdissection, chromosome sorting by flow cytometry is a high-throughput approach to purify a large amount of a particular chromosomes. Flow cytometric sorting of chromosomes has had a broad range of applications in genome research, and has been applied to DNA hybridization^23,24^, DNA libraries^25,26^, physical mapping^12,27,28^, and chromosome sequencing^1,5–11^. Chromosome sequencing was previously considered to be a time- and cost-effective method to sequence incompletely-annotated chromosomes^1^. Sequencing single chromosomes is more attractive because it can greatly simplify data analysis and reduce sequencing costs as compared to that with complex whole genome sequencing. Moreover, we believe that combined with the development of new sequencing technologies such as nanopore sequencing^29^, the flow cytometric sorting of chromosomes might be applied more widely and deeply for chromosome genome research. The ovine Y chromosome has not been assembled and annotated based on whole genome sequencing, and we expect to isolate ovine Y chromosomes by flow cytometric sorting and combine this with new sequencing technologies to analyze the ovine Y chromosome in the future.

The ovine chromosomes flow sorting was early reported in 1992, and only the first three large metacentric chromosomes and five other clusters could be resolved^30^. A high-resolution flow karyotype for sheep was obtained in 1997, from which nearly all chromosomes have been isolated and identified^19^. The bivariate flow karyotype of sheep obtained in this study was similar to that in Burkin’s work, and we distinguished and labeled each chromosome cluster in the bivariate flow karyotype of the sheep according that report. Moreover, most of the single chromosomes from sheep could be isolated in our work. We mainly isolated and sorted Y chromosomes by flow cytometric sorting and identified the flow-sorted Y chromosomes by FqRT-PCR, FISH, and NGS. This is the first report to identify the sorted ovine Y chromosomes by NGS. The alignment read results confirmed that we successfully enriched the ovine Y chromosome by flow cytometric sorting methods. Very low proportions of the reads were properly mapped to autosomal sequences in the ovine genome, except for chromosome 17 and chromosome X, to which 6.68% and 13.57% of reads aligned, respectively. This might be due to the presence of a very small number of highly similar sequences between chromosomes (including chromosome Y). In addition, this result was similar to NGS results of human Y chromosomes captured using magnetic streptavidin-beads, instead of through flow cytometry^31^. The discrepancy of the NGS results, as compared to those of this previous study, might be due to differing purities of target chromosomes during flow cytometric sorting. Chromosomes Y and X contain a homologous region called the pseudo-autosomal region (PAR), which is involved in sex chromosome pairing, recombination, and segregation during meiosis^32,33^. The ruminant PAR is estimated to be> 9 Mbp^32^, and thus, it is reasonable to assume that there are an adequate number of ovine Y chromosome reads successfully mapped to the X chromosome. Additionally, we found that 97.69% of the properly mapped reads in ovine chromosome 17 were located in the 70–72 Mb region, which includes a gene named *PRAME* (melanoma antigen preferentially expressed in tumors). The *PRAME* gene in the cattle genome is a multi-copied gene and was found to share high sequence similarity with the *PRAMEY* gene in cattle Y^34,35^. We speculated that the 6.68% of alignments from the sorted chromosomes with ovine chromosome 17 were mostly due to the occurrence of homologous of *PRAME* and *PRAMEY* in sheep. Moreover, 63.28% of reads were mapped to cattle Y chromosome sequences. The Y chromosomes in domestic sheep and cattle have different chromosome sizes and gene orders as a result of complex rearrangements^36^. However, the two Y chromosomes are highly similar since they were both derived from the ancient Bovidae Y chromosome. In conclusion, the alignments and distribution of the NGS reads of the sorted chromosomes indicate that the flow-sorted chromosomal fragments mainly originated from the ovine Y chromosome.

Data also reflected the purity of the flow-sorted chromosomes, and purity can vary widely based on flow cytometric sorting performance. In a previous report on the assembly of flow-sorted gorilla Y chromosomes, chromosome Y comprised ∼30% of sequenced flow-sorted material (the other material was postulated to be debris from other chromosomes)^1^. In another report in which the barley genome was investigated through chromosome flow cytometric sorting, NGS data confirmed that genes detected in a chromosome arm sequence dataset originating from the assigned source comprised> 95%^14^. The isolation of Y chromosomes of high purity facilitates the accumulation of materials for deep next- and third-generation sequencing of sorted ovine Y chromosomes and could result in the assembly of the entire ovine Y chromosome. Similar approaches have been successfully applied for assembly of the gorilla^1^ and human^5^ Y chromosomes.

Anyway, successful isolation of the target chromosomes provides the possibility to further study this chromosome. Cell synchronization effects and chromosome samples are critical to chromosome sorting results. The resolution of bivariate flow karyotypes was very sensitive to sample quality. The pH and NaCl concentration of the chromosome suspension, in addition to staining time, were factors that influenced the quality of flow karyotype resolution^15^. In addition, we found that the efficiency of cell cycle synchronization at the G2/M phase was closely related to the quality of chromosome samples. In our experiments, the proportion of cells at the G2/M phase, of all collected cells requiring lysis, was greater than 90%. And during the experiments, we found that the higher the synchronization efficiency of G2/M cells, the easier it is to obtain a flow karyotype with better resolution.

In summary, we described the optimal conditions for achieving highly efficient cell cycle synchronization at the G2/M phase using sheep primary skin fibroblasts. Metaphase cells enriched by this procedure and the resulted chromosome suspensions were suitable for flow cytometric sorting. Moreover, we successfully isolated and enriched the ovine Y chromosome and performed NGS on the sorted Y chromosomes. The fact that 46.49% of reads mapped to cattle Y chromosome sequences and 68.90% of the NGS reads were Y chromosome-related sequences indicated that the flow-sorted chromosomal fragments mainly originated from the ovine Y chromosome. To our knowledge, this is the first report verifying the purity and coverage of flow-sorted Y chromosomes from the ovine genome by NGS. This work lays a foundation for future research on sequencing and annotation of the ovine Y chromosome.

## Materials and Methods

### Establishment, purification, and culture of sheep primary skin fibroblasts

A small piece of ear tissue (10 × 10 mm) was cut from a 2.5-year-old healthy male Merino sheep. The tissue was cut into small pieces (1 mm^3^). Minced skin pieces and 10 mL of Dulbecco’s Modified Eagle’s Medium (DMEM) supplemented with 10% FBS and 1% antibiotics (penicillin and streptomycin) were put in 90 mm culture dishes. Culture dishes were placed in an incubator at 37 °C with a humid atmosphere containing 5% CO_2_, and the culture medium was changed every 2 days. When cell confluency reached 70–80%, the cells were treated with trypsin-EDTA (trypsin 0.10% and EDTA 0.02%) for 2 min for culture in the next passage.

### Cell-cycle synchronization

#### Observation of mitotic activity in sheep fibroblasts during recovery after double thymidine block treatment

Sheep primary skin fibroblasts were treated with thymidine (TDR; Sigma-Aldrich Corp, St. Louis, MO, USA) at a concentration of 2 mM for 16–18 h. The cells were then washed with PBS three times to remove thymidine and were then cultured in DMEM supplemented with 10% FBS and 1% antibiotics for an additional 8–10 h before the addition of thymidine (2 mM) for 16–18 h. Using this time point as the new starting point, cells were washed with PBS and harvested at 2-h intervals, for up to 24 h. The harvested cells were then fixed with cold ethanol (70%) for flow cytometric cell cycle analysis. Fibroblasts cultured in DMEM supplemented with 10% FBS and 1% antibiotics without any treatment were used as the control group.

#### Effect of nocodazole concentration

The effect of different concentrations of nocodazole (Sigma-Aldrich Corp, St. Louis, MO, USA) on inducing cell cycle arrest at the G2/M phase was evaluated by treating cells that had reached 70–80% confluency with nocodazole (50, 100, 200, 300, 400, 500, 700, and 1000 ng/mL) for 4–6 h. The cells were then washed with PBS and harvested, before being fixed for flow cytometric cell cycle analysis. Sheep primary skin fibroblasts cultured in DMEM supplemented with 10% FBS and 1% antibiotics without any treatment were used as the control group.

#### Double thymidine block and nocodazole/colchicine based synchronization

The double thymidine block procedure was performed as described previously herein. After the second treatment of thymidine (2 mM) for 16–18 h, cells were washed with PBS three times to remove thymidine. This time point was designated as 0 h. A group of cells were treated with nocodazole at a concentration of 100 ng/mL for 4 h for various time intervals (0–4 h, 1–5 h, 2–6 h, and 3–7 h) after thymidine removal. Another group of cells was treated with colchicine at a concentration of 100 ng/mL^37^ for 4 h at various time intervals (0–4 h, 1–5 h, 2–6 h, and 3–7 h). After 4 h of treatment with nocodazole (100 ng/mL) or colchicine (100 ng/mL), cells were washed with PBS, harvested, and fixed for flow cytometric cell cycle analysis. Passage-five sheep primary skin fibroblasts were used in this experiment. Fibroblasts without any treatment were used as the control group.

An additional two groups of cells were collected and analyzed. After double thymidine block and 2 h recovery treatment, a group of cells was treated with nocodazole (100 ng/mL) for 4 h, whereas another group was treated with colchicine (100 ng/mL) for 4 h. After a 4-h treatment with nocodazole (100 ng/mL) or colchicine (100 ng/mL), metaphase cells were suspended by patting the dishes gently and collecting them by centrifugation at 289 × g for 5 min. Cells collected after nocodazole treatment were designated TTNP and cells collected after colchicine treatment were designated TTCP.

#### Flow cytometric cell cycle analysis

Cell cycle analysis was performed according to methods described by Hashem *et al*.^38^. In brief, cells were stained with 0.5 mL PBS containing 40 μg/mL PI (propidium iodide; Sigma) and 0.3 mg/mL RNase A at 37 °C for 30 min. The stained cells were analyzed using a BD FACS Calibur flow cytometer at 488 nm. For each sample, 20,000 events were recorded and histograms were plotted. The percentages of cells within various phases of the cell cycle were calculated using FlowJo software (Treestar, Ashland, OR).

### Statistical analysis

Each treatment group was repeated at least three times. One-way analysis of variance (ANOVA) was used to analyze statistical differences among groups. *P* < 0.05 was considered statistically significant.

### Accumulation of metaphase cells

Sheep primary skin fibroblasts were treated with thymidine at a concentration of 2 mM for 16–18 h. After the first TDR (2 mM) treatment, cells were then washed with PBS three times to remove thymidine and were further incubated for 8–10 h in DMEM supplemented with 10% FBS and 1% antibiotics. Cells were then treated with thymidine (2 mM) for 16–18 h for a second time. After the second TDR (2 mM) treatment, cells were washed with PBS three times to remove thymidine and were incubated once again in DMEM supplemented with 10% FBS and 1% antibiotics. After 2 h, nocodazole (100 ng/mL) was added and the cells were further incubated for 4–6 h. The supernatant was collected from the culture dishes after patting the dishes lightly. Metaphase cells in the supernatant were collected through centrifugation at 289 × *g* for 5 min. Then, 1.0 × 10^5^ cells were taken from these collected cells and were separated and fixed for flow cytometric cell cycle analysis. All remaining collected cells were used for the isolation of metaphase chromosomes.

### Isolation of metaphase chromosomes

The protocol used to isolate metaphase chromosomes for flow cytometric sorting was essentially based on an earlier report by Yang *et al*.^18^. Briefly, cells in the metaphase stage were gently resuspended in hypotonic solution (75 mM KCl, 0.2 mM spermine, 0.5 mM spermidine, 10 mM MgSO_4_·7H_2_O, pH 8.0) for 30 min at RT. The swollen cells were centrifuged at 289 × *g* for 5 min. The cell pellet was then resuspended gently in 1 mL of ice-cold polyamine isolation buffer (15 mM tris, 2 mM EDTA, 0.5 mM EGTA, 80 mM KCl, 3 mM dithiothreitol, 0.25% triton X-100, 0.2 mM spermine, 0.5 mM spermidine, pH 7.5). After 10 min of incubation on ice, the suspension was vortexed for 30 s and centrifuged at 201 × *g* for 2 min. The suspension was filtered through a 20-μM mesh filter (Celltrics, Partec, Münster, Germany). Then, 5 μL of this chromosome suspension was mixed with 5 μL of PI (100 μg/mL, Sigma) on a microscope slide and covered with a coverslip. The slide was monitored under a fluorescence microscope to check for “single floating chromosomes” in suspension.

### Flow karyotype analysis of ovine chromosomes and isolation of Y chromosomes

The chromosome suspension was stained overnight with 5 μg/mL of Hoechst 33258 (Sigma), 40 μg/mL of Chromomycin A3 (Sigma), and 10 mM MgSO_4_. Then, 10 mM of sodium citrate and 25 mM of sodium sulfite were added to the stained chromosome suspension at least 1 h before flow cytometric analysis. Bivariate flow analysis was performed using a FACSAria SORP Plus dual laser flow cytometer (Becton Dickinson).

### Verification of Y chromosomes sorted by flow cytometry

The purity of the flow-sorted Y chromosome suspension was assessed by fluorescence quantitative real-time polymerase chain reaction (FqRT-PCR) of known specific segments of the Y chromosome, as well as fluorescence *in situ* hybridization (FISH). Sorted Y chromosomes were used as templates for amplification with REPLI-g Single Cell Kit (QIAGEN). The reaction products were used as templates for FqRT-PCR and the preparation of painting probes for FISH.

### Fluorescence quantitative real-time PCR

Although there was no Y chromosome reference genome sequence for sheep, the male-specific region of the Y chromosome (MSY) has been used for Y chromosome haplotypes and sex-determination^39,40^. Primers for FqRT-PCR used in this study are shown in Supplementary Table S4. Ovine genomic DNA was the template control for the FqRT-PCR, and 100 ng/μL of DNA was used. Samples were denatured at 95 °C for 1 min followed by 40 cycles of 95 °C for 5 s, 60 °C for 20 s, and 72 °C for 20 s.

### Preparation of Y chromosome painting probes and FISH

The Y chromosome paints were directly labeled with biotin using the BioPrime DNA labeling system (Invitrogen) and hybridized onto metaphase spreads of sheep primary skin fibroblasts. The FISH procedure was performed as described by Jia *et al*.^41^. Fluorescent signals were detected using a confocal microscope (OLYMPUS FV1000) and images were captured with FV10-ASW software (Olympus Optical Co., Tokyo, Japan).

### Whole genome amplification (WGA) and NGS of Y chromosomes from sheep

Flow-sorted Y chromosomes were subsequently used as templates for WGA performed with the looping-based amplification cycle (MALBAC) Kit (Yikon Genomics, China). Concentrations and sizes of the WGA products were detected with a NanoDrop 2000 spectrophotometer and agarose gel electrophoresis. The WGA products were subsequently processed for the construction of libraries and NGS. The sequencing library was constructed using the TruSeq DNA Sample Preparation Kit (Illumina, USA) according to the manufacturer’s instructions. Sequencing was performed with an Illumina HiSeq. 4000 platform (Illumina, USA), and 150-bp paired-end reads were generated for the following analysis.

### Aligning reads to the ovine genome and the cattle Y chromosome

Raw reads were first trimmed according to Trimmomatic *et al*. to identify clean data by discarding adapter sequences and low quality reads^42^. The BWA mem^43^ algorithm with default options was used to align clean reads against the sheep reference genome (OAR v4.0, GenBank accession: GCA_000298735.2), in addition to the Y chromosome from cattle (*Bos taurus*; GenBank accession: CM001061.2). SAMtools^44^ was used to collect mapping statistics, the number of mapped reads, and the proper mapping rate from the resulting mapped files.

### Ethics approval and consent to participate

All animal experiments were approved by the Beijing Administration Committee of Laboratory Animals and conducted in accordance with the China Agricultural University (CAU) Institutional Animal Care and Use Committee guidelines (ID: SKLAB-B-2010–003). The protocols for animal use and experimentation were approved by the Beijing Association for Science and Technology (approval ID SYXK [Beijing] 2007–0023) and were in compliance with the Beijing Laboratory Animal Welfare and Ethics guidelines.

## Supplementary information

Supplementary information.

## Data Availability

The raw reads of Next-Gen sequence from the amplified, sorted ovine Y DNA samples will be available on the NCBI SRA database and apply for delayed disclosure.
