# Supplementary material for: Highly efficient synchronization of sheep skin fibroblasts at G2/M phase and isolation of sheep Y chromosomes by flow cytometric sorting
Source: Sci Rep. 2020 Jun 18;10:9933. doi: 10.1038/s41598-020-66905-x (PMC7303189; doi:10.1038/s41598-020-66905-x)
Supplement: Supplementary file 1 — Supplementary information. [file 41598_2020_66905_MOESM1_ESM.docx]

**Supplementary Information**

**Highly efficient synchronization of sheep skin fibroblasts at G2/M phase and isolation of sheep Y chromosomes by flow cytometric sorting**

Yanzhu Yao^1^, Yuanyuan Zhang^1^, Wangsheng Liu^2^, Xuemei Deng^1*^

^1^Key Laboratory of Animal Genetics, Breeding and Reproduction of the Ministry of Agriculture & Beijing Key Laboratory of Animal Genetic Improvement, Beijing 100193, China, China Agricultural University, Beijing 100193, China;

^2^Department of Animal Science, Center for Reproductive Biology and Health, College of Agricultural Sciences, Pennsylvania State University, University Park, Pennsylvania 16802, USA

*Corresponding author. E-mail address: deng@cau.edu.cn (X.D).

**Corresponding author: Xuemei Den**g. Key Laboratory of Animal Genetics, Breeding, and Reproduction of the Ministry of Agriculture & Beijing Key Laboratory of Animal Genetic Improvement, China Agricultural University, Beijing 100193, China;

[Phone number]+86-010-62733933

[Email address] deng@cau.edu.cn

**Supplementary Figure S1**


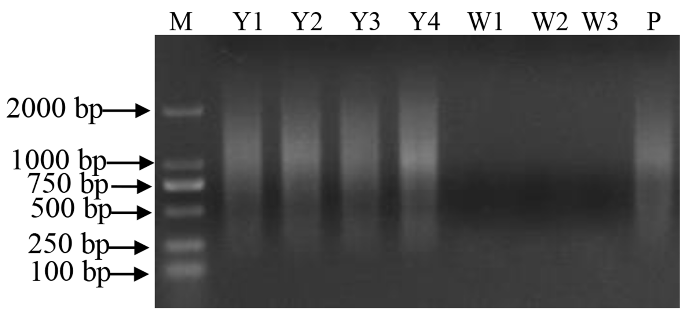


**Supplementary Figure S1.** Detection of WGA products by agarose gel electrophoresis. Y1, Y2, Y3 and Y4: WGA products of Y chromosome DNA; W1, W2 and W3: water as negative control; P: sheep genomic DNA as a positive control.

**Supplementary Figure S2**


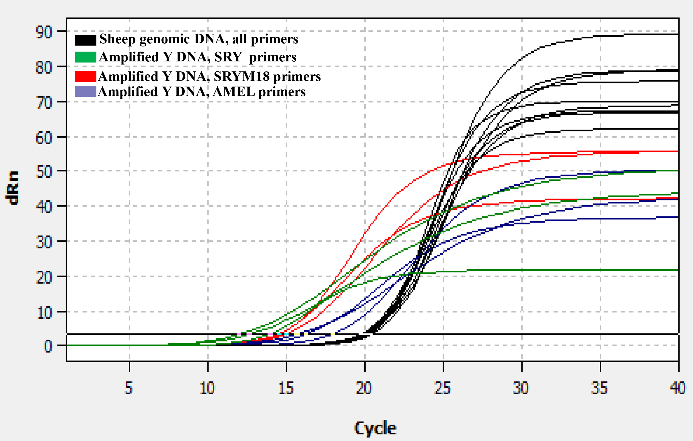


**Supplementary Figure S2.** Verification of flow sorted Y chromosomes by Real-time PCR amplification. CT values of all primers using the amplified Y chromosome DNA as templates were lower than the CT values of all primers using sheep genomic DNA as templates.

**Supplementary Table S1**. Products of WGA (MALBAC method) of flow-sorted Y chromosome DNA

| Sample name | Concentration of products (ng/ul) | volume of products (ul) |
| --- | --- | --- |
| Y1 | 38.8 | 50 |
| Y2 | 46.8 | 50 |
| Y3 | 57.6 | 50 |
| Y4 | 62.6 | 50 |

**Supplementary Table S2.** Ct values of Q-PCR for Verification of Y chromosomes

| sequences | CT values of sheep genomic DNA | CT values of amplified Y DNA |
| --- | --- | --- |
| SRY | 19.98±0.18^a^ | 14.61±0.19^b^ |
| AMEL | 20.17±0.12^a^ | 16.62±0.92^b^ |
| SRYM18 | 19.90±0.05^a^ | 12.85±0.73^b^ |

Within a row, different superscripts (a, b) are significantly different (*P* < 0.05).

**Supplementary Table S3.** Proportion of reads that mapped to each chromosome

| chromosome | Properly mapped reads/Total reads (%) |
| --- | --- |
| chr1 | 0.86 |
| chr2 | 1.18 |
| chr3 | 0.77 |
| chr4 | 0.27 |
| chr5 | 0.25 |
| chr6 | 0.30 |
| chr7 | 0.39 |
| chr8 | 0.17 |
| chr9 | 0.26 |
| chr10 | 0.20 |
| chr11 | 0.26 |
| chr12 | 0.26 |
| chr13 | 1.10 |
| chr14 | 0.27 |
| chr15 | 0.27 |
| chr16 | 0.17 |
| chr17 | 6.68 |
| chr18 | 0.25 |
| chr19 | 0.74 |
| chr20 | 0.74 |
| chr21 | 0.17 |
| chr22 | 0.22 |
| chr23 | 0.38 |
| chr24 | 0.11 |
| chr25 | 0.61 |
| chr26 | 0.14 |
| ChrX | 13.57 |
| MT | 0.00 |
| unknown | 0.52 |
| unmapped | 68.90 |

**Supplementary Table S4.** Primers used in fluorescence quantitative real-time polymerase chain

reaction (FqRT-PCR) for verification of Y chromosomes.

| Sequence name | Primer sequence (5' - 3') | PCR amplification length |
| --- | --- | --- |
| AMEL | CAGCCAAACCTCCCTCTGC | 217 bp |
|  | CCCGCTTGGTCTTGTCTGTTGC |  |
| SRY | AGCTCCAGAATATTTCACTGACCT | 130 bp |
|  | GAAGGCAAATGCAGAGACAA |  |
| SRYM18 | GGCATCACAAACAGGATCAGCAAT | 106 bp -145 bp |
|  | GTGATGGCAGTTCTCACAATCTCCT |  |
